# Compassion Is Not a Benzo: Distinctive Associations of Heart Rate Variability With Its Empathic and Action Components

**DOI:** 10.3389/fnins.2021.617443

**Published:** 2021-03-12

**Authors:** Maria Di Bello, Cristina Ottaviani, Nicola Petrocchi

**Affiliations:** ^1^Department of Psychology, Faculty of Medicine and Psychology, Sapienza University of Rome, Rome, Italy; ^2^Functional Neuroimaging Laboratory, IRCCS Santa Lucia Foundation, Rome, Italy; ^3^Department of Economics and Social Sciences, John Cabot University, Rome, Italy

**Keywords:** compassion, action, heart rate variability, empathic engagement, empathic sensitivity

## Abstract

Recent studies have linked compassion with higher vagally mediated heart rate variability (vmHRV), a measure of parasympathetic activity, and meta-analytic evidence confirmed significant and positive associations. Compassion, however, is not to be confused with soothing positive emotions: in order to engage in actions aimed to alleviate (self or others) suffering, the pain should resonate, and empathic sensitivity should be experienced first. The present study examined the association between vmHRV and the empathic sensitivity and action components of trait and state compassion. To do so, several dispositional questionnaires were administered and two videos inducing empathic sensitivity (video 1) and compassionate actions (video 2) were shown, while the ECG was continuously recorded, and momentary affect was assessed. Results showed that (i) scores on subscales assessing the empathic component of trait compassion were inversely related to resting vmHRV; (ii) vmHRV decreased after video 1 but significantly increased after video 2. As to momentary affect, video 1 was accompanied with an increase in sadness and a decrease in positive affect, whereas video 2 was characterized by an increase in anger, a parallel decrease in sadness, and an increase (although non-significant) in positive affect. Overall, present findings support the notion that it is simplistic to link compassion with higher vmHRV. Compassion encompasses increased sensitivity to emotional pain, which is naturally associated with lower vmHRV, and action to alleviate others’ suffering, which is ultimately associated with increased vmHRV. The importance of adopting a nuanced perspective on the complex physiological regulation that underlies compassionate responding to suffering is discussed.

## Introduction

Over two decades, studies have shown the health promoting influences of compassion (Pace et al., 2009, 2013; Seppala et al., 2012; Slavich and Cole, 2013; Yarnell and Neff, 2013; Zessin et al., 2015), fostering the development of different compassion-focused interventions and scientific research on the nature of therapeutic process (Kirby, 2016; Ferrari et al., 2019; Gilbert, 2019). The aim of the present study was to investigate the physiological signature (measured by vagally mediated heart rate variability; vmHRV) of the specific components of trait and state compassion.

Compassion has been defined as a sensitivity to suffering in self and others with a commitment to try to alleviate and prevent it (Gilbert, 2019); it does not refer to a positive emotional experience but to a suite of concrete prosocial behaviors, and may include positive emotional states such as kindness, empathy, generosity, and acceptance (Weidman and Tracy, 2020). Conversely, kindness is intended to create the conditions for happiness and prosperity; it does not require any sensitivity to and analysis of suffering (Gilbert et al., 2019).

As an evolved prosocial motivation, compassion requires complex social processing systems and evolved competencies which allow approach and engagement with distress signals to help alleviate the distress, such as, a sensitive awareness of suffering, empathic awareness, distress tolerance, a non-judgmental attitude, and the willingness to develop specific skills to enact the motive (knowing intentionality) (Gilbert, 2017a, 2019). This set of competencies does not translate compassion into an automatic response to suffering, but into a complex motivational process which guides the individual to be sensitive and receptive to signals of suffering, as opposed to trying to avoid or suppress them, and understand what is the most helpful thing to do in the specific circumstance.

Over the past decade, the psychophysiological perspective has contributed significantly to our understanding of compassion in an effort to provide unique insights into its nature and processes. A specific role of the 10th cranial nerve, namely the vagus, has been highlighted in compassion-related processes (Thayer et al., 2012; Porges, 2017; Petrocchi and Cheli, 2019). Efferent vagal fibers exert parasympathetic control of the heart. An indirect and non-invasive measure of vagal modulation of the heart is vmHRV, a measure of the variability of the time periods between adjacent heartbeats, resulting from the dynamic interplay between the parasympathetic and the sympathetic nervous systems (Task Force of the European Society of Cardiology and the North American Society of Pacing and Electrophysiology, 1996). In particular, high tonic vmHRV is a measure of robust parasympathetic control on the heart and appears to reflect the degree to which the prefrontal cortex provides context-appropriate control over the periphery. On the other hand, phasic vmHRV suppression represents the withdrawal of cardiac vagal control and the activation of the defensive systems (Thayer et al., 2009, 2012).

Recent studies (Svendsen et al., 2016, 2020; Luo et al., 2018) and meta-analytic evidence (Di Bello et al., 2020) showed that both state (i.e., induced) and trait (i.e., dispositional) compassion, toward oneself and others, are related to higher vmHRV (Di Bello et al., 2020). Additionally, compassion-focused practices can improve vmHRV (Rockliff et al., 2008; Arch et al., 2014; Matos et al., 2017; Petrocchi et al., 2017).

Previous studies, however, ignored a crucial ingredient of compassion, that is whether the ability to pay sensitive attention to suffering and effectively engage with it is accompanied by the intention and capacity to start the response process (an appropriate action) to alleviate the suffering (Gilbert, 2017b; Kirby et al., 2019; Gilbert, 2020). If both these components are taken into account, a non-linear association between compassion and vmHRV is expected (Miller, 2018). For example, attentional sensitivity and tolerance to other’s distress may be associated with a decrease in vmHRV. Indeed, individuals with lower vmHRV show reduced ability to inhibit attention to affectively significant cues (Park et al., 2013) and the anticipation of social stress has shown to reduce vmHRV in individuals with high self-compassion (Luo et al., 2018). Additionally, in response to observation of others’ emotional expressions, lower resting vmHRV was associated with higher activation in the mirror neuron system (Miller et al., 2019).

Indeed, a phasic decrease in vmHRV could be considered as a signature of the empathic engagement with others’ suffering and it is the prerequisite to be touched by others’ suffering and subsequently act to alleviate it (Miller, 2018; Gilbert, 2019; Steffen et al., 2020). Heart rate (and not vmHRV) has shown to increase more during compassion meditation than during neutral states, and HR increases are correlated with BOLD signal in the right middle insula (Lutz et al., 2008), suggesting that compassion enhances the emotional and somatosensory brain representations of others’ emotions, amplifying the saliency of emotional stimuli.

Based on such evidence, compassion is expected to be first associated with reduced vmHRV to reflect empathic engagement with suffering, and then by increased vmHRV when the appropriate helpful action is performed. Indeed, in a pilot uncontrolled study, self-compassionate writing has been associated with a significant decrease in vmHRV during the task, and a significant increase in vmHRV during recovery (Steffen et al., 2020).

To date, the physiological signatures of each of the different components of compassion have not been thoroughly investigated, neither when dispositional (trait) nor when induced (state) compassion was examined. The study aimed to fill this gap, assessing resting vmHRV, the specific components of trait compassion, and vmHRV responses to and recovery from videos evoking the different components of compassion (i.e., empathic sensitivity and compassionate action). We hypothesized: (i) the association between tonic vmHRV and trait compassion to be negative for the empathic component and positive for the engagement component of compassion; (ii) to see a vmHRV decrease in response to a video eliciting empathetic sensitivity toward others’ suffering, and a vmHRV increase in response to a video depicting intentional actions of giving help. We expected vmHRV reactivity to and recovery from such videos to be moderated by self and other-oriented trait compassion.

## Materials and Methods

### Participants

The sample consisted of 45 students [88% female, mean age = 20.5 (1.05) years] of an American university based in Rome, Caucasian, and English speaking. Three subjects were not included in the analyses due to unreliable physiological measures (*N* = 42, 88% female). Exclusionary criteria were self-reported major psychiatric or cognitive problems, organic illnesses, substance abuse, cardiovascular disease, use of drugs/medications affecting cardiovascular function, body mass index >30 kg/m^2^, menopause, use of oral contraceptives during the previous 6 months, pregnancy or childbirth within the last 12 months. The protocol was approved by the local Ethics Committee.

### Procedure and Design

**FIGURE 1:**
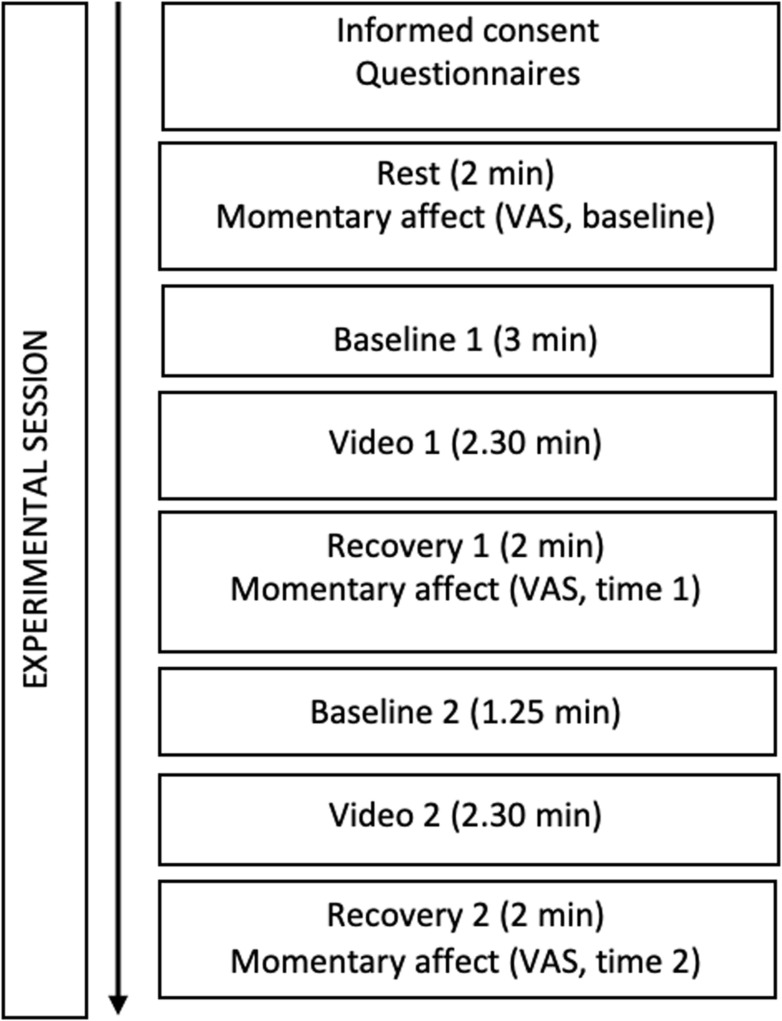
Flowchart illustrating the experimental procedure.

The experiment was conducted as a repeated measures within-subjects experimental design (Figure 1). Written informed consent was provided. Participants were asked to refrain from drinking caffeinated or alcoholic beverages or exercising in the 2 h prior to the session. Data on socio-demographic information, medical background and currently prescribed medications were first collected. Then, several self-report trait measures of compassion were administered. Participants were asked to rest for a 5-min baseline ECG assessment, during which they rated their momentary affect by visual analog scales (VAS, baseline). Next, a 2.30-min video representing an “empathic sensitivity” condition was shown. This was followed by a post-video recovery assessment (2 min), during which the VAS were administered again (time 1). To collect a second baseline, participants were then instructed to relax for another 1.25 min. Subsequently, a second video representing a “compassionate action” condition was broadcast for 2.30 min, followed by a post-video recovery assessment (2 min), during which participants had to rate again their current affect by VAS (time 2). The videos are available from the last author on request.

### Stimulus Materials

Instructions were displayed on a computer monitor, while a guide-voice signaled participants to relax at baseline and gave indications on how to rate the experienced feelings on the VAS, and how to watch the videos. A pilot study was conducted to make sure that each video elicited one of the two specific components of compassion: empathic sensitivity by video 1 and intention to perform helpful actions by video 2. In video 1, others’ suffering scenes were presented along with brief sentences describing the thoughts of each suffering individuals, aimed to facilitate the empathic attunement with them. Video 2 depicted scenes of a person actively engaged in giving help and support to suffering others. The participants had to pay attention, allowing themselves to experience thoughts and feelings, while taking the perspective of the person who gives help.

### Measures

#### Socio-Demographic and Personal Information

Socio-demographic and personal information, included sex, age, height, and weight, and physical activity habits.

#### Compassionate Engagement and Action Scales

Compassionate Engagement and Action Scales (CEAS; Gilbert et al., 2017) encompasses subscales assessing three “flows” of compassion (for others, from others, and self-compassion). Respondents are asked to think about distressing situations and rate how each sentence applies to them. For each scale, a total score and two subscales were calculated: Engagement and Actions. For the Compassion for Self scale, two subdimensions were analyzed in the Engagement subscale: Sensitivity to Suffering, and Engagement with Suffering.

#### Compassionate Love for Humanity Scale

Compassionate Love for Humanity Scale (Sprecher and Fehr, 2005) is a 21-item scale designed to measure the compassionate attitude for strangers when they are most in need.

#### Compassionate and Self-Image Goals Scale

Compassionate and Self-Image Goals Scale (Crocker and Canevello, 2008) consists of 13 items. Seven items assess compassionate goals, and six items assess self-image goals. The average for each subscale was calculated, with higher scores indicating higher interpersonal goals.

#### Visual Analog Scales – VAS

At baseline and after each video (time 1 and time 2), participants rated their current levels of affective states (sad, angry, happy, anxious, calm, strong, weak, content, relieved, self-critical) on several 5-point VAS.

#### Physiological Measures

Interbeat intervals were continuously recorded throughout the experimental session using the Firstbeat Bodyguard 2 with a standard electrode configuration. Time (root mean square successive difference; RMSSD) and frequency-domain (high-frequency HRV; HF-HRV) vmHRV measures were calculated. VmHRV analysis was performed using Kubios HRV software (Tarvainen et al., 2014). Artifacts and ectopic beats were corrected using a threshold-based correction.

### Data Analyses

The data were analyzed using IBM SPSS Statistics version 25 and Mplus 5.1. To evaluate the effects of dispositional variables (socio-demographic factors and trait measures) on the dependent variables, Pearson correlations were performed between BMI, physical activity, trait questionnaires, and vmHRV. Sex differences were evaluated by Student’s *t*-test. The variables that resulted to be significantly associated with vmHRV measures were included in the subsequent analyses as covariate.

Following existing recommendations (Laborde et al., 2018), to determine whether the two videos induced a different physiological response, we computed reactivity and recovery scores by subtracting the baseline from each video-phase and the video-phase from each recovery-phase, respectively. A 2 × 2 mixed-model ANOVA was conducted on vmHRV with time (Reactivity; Recovery) as within-subjects variable, and video (video 1, video 2) as the between-subjects factor. Next, post hoc comparisons were used to identify significant differences between means (Reactivity to video 1 and 2, Recovery from video 1 and 2).

Moderation analyses were executed using the Hayes (2012) PROCESS macro version 3.5 to test conditional effects by trait variables on physiological responses. Specifically, Self-compassion and Compassion to Others scales of the CEAS were tested as moderators of the size of the effect of videos on physiological responses (Recovery and Reactivity). Both subscales (Engagement and Action) were tested for their moderating effects.

An exploratory factor analysis on VAS has been then conducted to identify associations within self-reported momentary affect variables. We used Principal Component Analysis (PCA) and Varimax orthogonal rotation as methods to extract the factors. Cattell’s (1966) scree test was used as decision rule for identifying the number of factors to retain. Then, a structural equation model (SEM) was run using Mplus 5.1 (Muthén and Muthén, 2008) to conduct a set of confirmatory analyses to assess the relative fit of the three-factors model that emerged from the exploratory factor analysis (Positive Affect, low arousal Negative Affect, high arousal Negative Affect). A non-significant chi-square test was considered as evidence of good fit. Following the recommendations of Kline (1998), multiple indexes were used to evaluate the goodness of fit of the model. These included the comparative fit index (CFI), Tucker–Lewis index (TLI), and standardized root-mean-square residual (SRMR). Acceptable fit was defined as CFI and TLI values of 0.90 or greater and SRMR of 0.05 or less.

To determine if the two videos induced different emotional responses, a series of repeated-measure analysis of variance (ANOVA) was performed on factorial scores with time (baseline, video 1, video 2) as the within-subjects factor. Effect sizes were calculated using partial eta squares (η^2^_*p*_), with η^2^*_*p*_* = 0.01 referring to a small effect size, 0.06 to a medium effect size and 0.14 to a large effect size (Tabachnick and Fidell, 2013).

## Results

Given the high correlation between the two vmHRV indices (rMSSD and HF-HRV; *r* = 0.93 for baseline 1; *r* = 0.95 for baseline 2; all *p*s < 0.0001), all the analyses have been performed on HF-HRV only. Correlation analysis showed negative and significant associations between HF-HRV and the following trait measures: Self Compassion total score (*r* = −0.324; *p* = 0.036), Sensitivity to Suffering-Self-Compassion component (*r* = −0.307; *p* = 0.048), Compassion to Others total score (*r* = −0.407; *p* = 0.007), and Compassion to Others-Engagement component (*r* = −0.445; *p* = 0.003).

**FIGURE 2:**
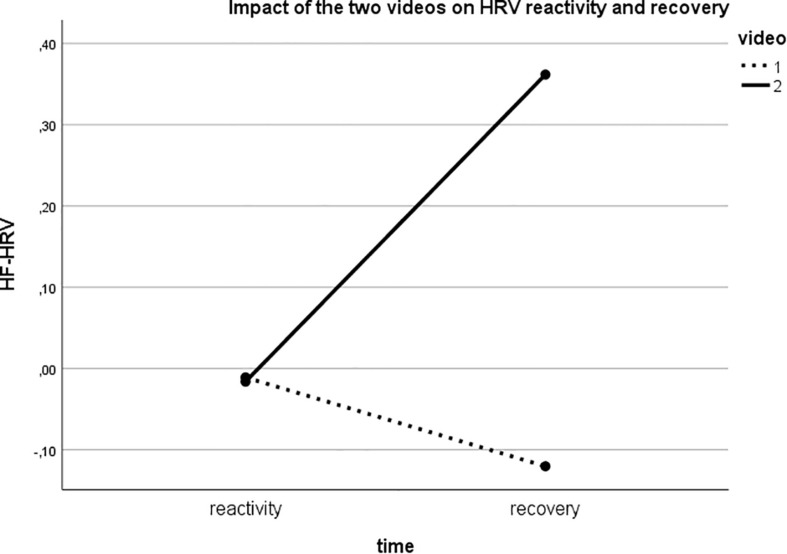
Significant Time by Video interaction showing that while reactivity to the two videos did not differ, high-frequency heart rate variability (HF-HRV) increased during recovery from Video 2 (action component of compassion) but decreased during recovery from Video 1 (empathic sensitivity component of compassion).

In line with our main hypotheses, the mixed-model ANOVA revealed a main effect of Video for HF-HRV [*F*_(__1_,_42__)_ = 7.465; *p* = 0.009; η*^2^_*p*_* = 0.151] and significant Time × Video interaction [*F*_(__1_,_42__)_ = 6.733; *p* = 0.013; η*^2^_*p*_* = 0.138] (Figure 2). Reactivity did not differ between the two videos (*t* = 0.148; *p* = 0.883); however, recovery from video 2 showed a significant increase in HF-HRV compared to recovery from the video 1 (*t* = 2.553; *p* = 0.003). Given the lack of differences in reactivity to the two videos, moderation analyses were performed only on recovery values.

**FIGURE 3:**
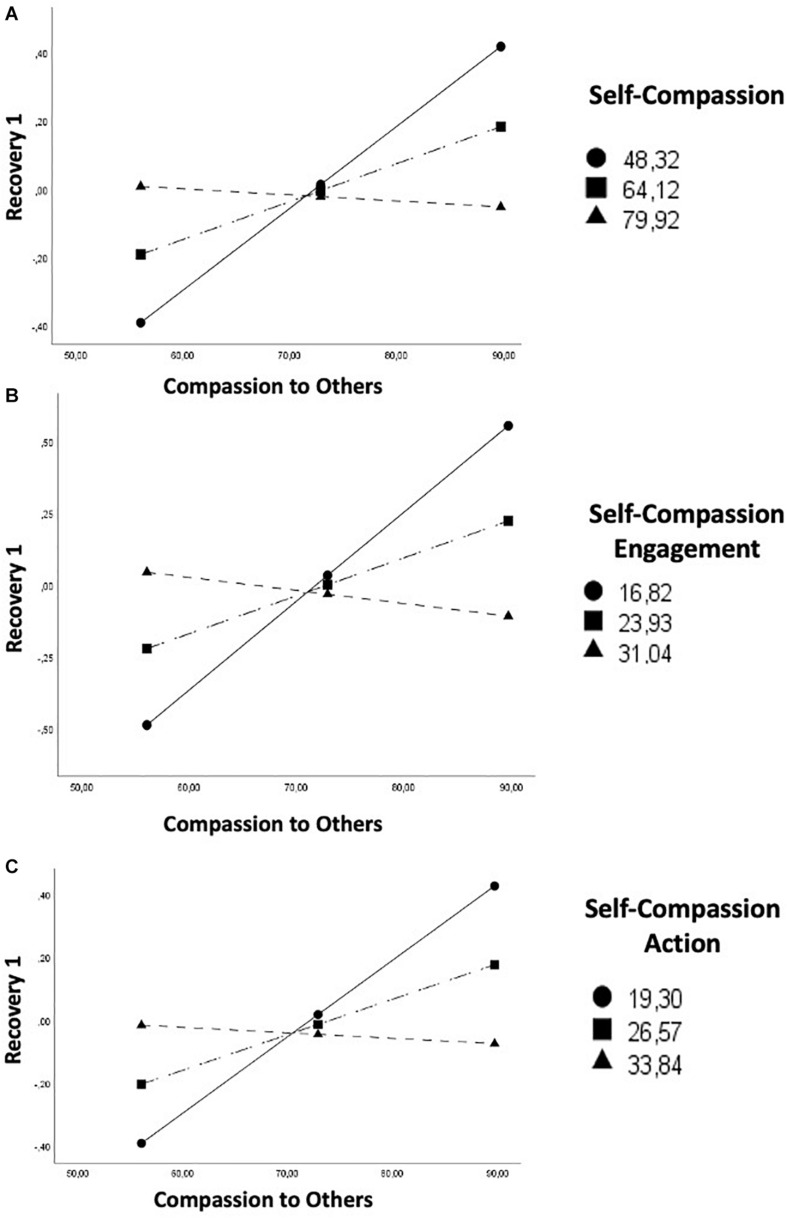
Self-compassion [total score, **(A)**; Engagement subscale, **(B)**; and Action subscale, **(C)**] moderates the association between Compassion for Others and High-Frequency Heart Rate Variability (HF-HRV) recovery from Video 1.

Multiple regression analysis showed no significant moderating effects on HF-HRV recovery from video 2 [*R*^2^ = 0.1447; *F*_(__3_,_38__)_ = 2.1435; *p* = 0.1108], whereas a significant effect of Compassion to Others (β = 0.0111; *SE* = 0.0044, *p* = 0.0153) but not of Self Compassion (β = −0.0011; *SE* = 0.0046; *p* = 0.8090) emerged for Recovery from video 1, with a significant interaction effect (β = −0.0008; *SE* = 0.0002; *p* = 0.0005). Overall, the regression model explained the 50% of the observed variance of HF-HRV recovery from Video 1 [*F*_(__3_,_38__)_ = 12.79; *p* = 0.0001]. The analysis of simple slopes of the interaction effect highlighted that the relationship between Compassion to Others and Recovery 1 was significant for low (β = 0.0241; *SE* = 0.0047; *p* = 0.0001) and medium Self-Compassion scores (−1 × *SD*) (*b* = 0.0111; *SE* = 0.0044; *p* = 0.0153), while for high scores the relationship was negative and non-significant (β = −0.0018; *SE* = 0.0062; *p* = 0.7761) (Figure 3A).

Results also revealed conditional effects of the Engagement with Suffering [*R*^2^ = 0.5247; *F*_(__3_,_38__)_ = 13.9851; *p* = 0.0001; interaction effect: β = −0.0025; *SE* = 0.0006; *p* = 0.0002] (Figure 3B) and Action [*R*^2^ = 0.5069; *F*_(__3_,_38__)_ = 13.0196; *p* = 0.0001; interaction effect: β = −0.0018; *SE* = 0.0005; *p* = 0.0003] (Figure 3C) subscales of Self Compassion on HF-HRV recovery from video 1. No significant moderating effect emerged by the Sensitivity to Suffering subscale.

The Scree plot based on exploratory factor analysis of VAS scores at baseline, revealed that the first 3 factors explained 69% of the variance in the data. The items exhibited loading values of ≥0.5, suggesting significant contribution. Thus, we selected the three-factor solution for further analyses. A three-factor model composed by (a) POSITIVE AFFECT (happy, calm, strong, content, relieved); (b) low arousal NEGATIVE AFFECT (sad, weak, anxious); and (c) high arousal NEGATIVE AFFECT (angry, self-critical; Table 1) exhibited good fit. Coherently with modification indices, and according to the theoretical framework behind the present study (Gilbert, 2020), we have opted for a Model including the following three factors: (a) POSITIVE AFFECT (happy, calm, strong, content, relieved); (b) low arousal NEGATIVE AFFECT (sad, weak); (c) high arousal NEGATIVE AFFECT (angry, self-critical, anxious).

**TABLE 1:** Correlations, means and standard deviations for visual analog scales (VAS) and goodness-fit indexes for the confirmatory factor analysis.

Subtest	1	2	3	4	5	6	7	8	9	10
M	1.62	1.24	3.10	2.29	3.02	2.98	1.93	3.36	2.10	2.67
SD	0.795	0.692	0.821	1.293	1.199	0.749	1.113	0.932	1.078	1.223
1. Sad	1									
2. Angry	0.346*	1								
3. Happy	–0.167	−0.385*	1							
4. Anxious	0.298	0.195	−0.417**	1						
5. Calm	–0.016	–0.183	0.394**	−539**	1					
6. Strong	–0.016	0.011	0.480**	−0.194	0.191	1				
7. Weak	0.575**	0.213	−0.393*	0.625**	–0.200	−0.324*	1			
8. Content	–0.273	−0.362*	0.751**	−0.532**	0.407**	0.292	−0.374*	1		
9. Relieved	–0.071	–0.031	0.458**	−0.370*	0.545**	0.487**	–0.198	0.475**	1	
10. Self-Critical	0.468**	–0.163	–0.138	0.401**	0.022	–0.009	0.466**	–0.192	0.025	1

**Model**	**χ*2***	***df***	***p***	**CFI**	**TLI**	**SRMR**				

**1**	*n*.*c*.									
**2**	12.591	32	0.99	1.000	16.058	0.058				
**3**	7.513	32	1.00	1.000	19.998	0.042				

*p < 0.05; **p < 0.01. n.c., no convergence.

**FIGURE 4:**
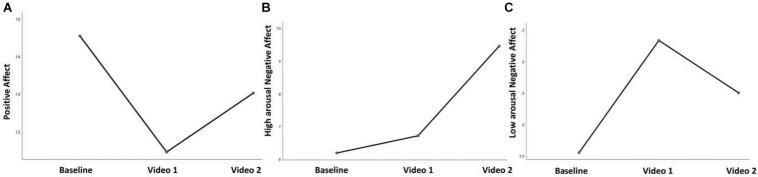
Self-reported changes in Positive Affect **(A)**, high-arousal Negative Affect **(B)**, and low-arousal Negative Affect **(C)** in response to two videos.

The repeated measures ANOVA revealed the following significant changes in response to the two videos: Positive Affect [*F*_(__2_,_82__)_ = 12.106; *p* = 0.0001; η*^2^_*p*_* = 0.228; Figure 4A], high arousal Negative Affect [*F*_(__2_,_82__)_ = 45.798; *p* = 0.0001; η*^2^_*p*_* = 0.528; Figure 4B], and low arousal Negative Affect [*F*_(__2_,_82__)_ = 19.982; *p* = 0.0001; η*^2^_*p*_* = 0.328; Figure 4C]. Participants specifically reported to be sadder (*p* = 0.0001, *M* increase = 179%), and less happy and strong (*p* = 0.0001, *M* decrease = 309%) in response to video 1, whereas in response to video 2 they reported to be angrier, and more self-critical compared to baseline (*p* = 0.0001, *M* increase = 328%) and to video 1 (*p* = 0.0001, *M* increase = 273%) but less sad compared to video 1 (*p* = 0.014, *M* decrease = 83%). An increase in Positive Affect in response to video 2 also emerged, although not statistically significant (Figure 4).

## Discussion

The present investigation aimed to examine the association between vmHRV and the specific components of trait and state (induced) compassion, namely empathic sensitivity and compassionate action. When the dispositional tendency to engage in compassion was examined, data evidenced that only the Compassionate Engagement Actions Scale (CEAS) showed associations with vmHRV. Notably, significant negative associations emerged between resting HF-HRV and dispositional compassion. This finding is not completely unexpected. Indeed, most of the studies that investigated the connection between compassion and vmHRV, used different measurement tools, which reflect different definitions of compassion and do not take into account the subdivision of engagement and action as fundamental components of compassion motivation (Gilbert, 2020). In line with our view that distress sensitivity and awareness involve specific physiological competencies for enabling emotional resonance to emotional pain, HF-HRV showed distinct negative associations with the element of empathic sensitivity (engagement) for both self- and other-oriented compassion. Notably, the sensitivity to suffering is an essential attribute of compassion that involves being responsive to one’s own suffering or to other people’s emotions (rather than activate defense mechanisms and avoidance), and perceiving when they need help (Gilbert, 2019).

Consistently, recent findings show that a more efficient shifting of attention from affective to non-affective aspects of negative information was related to lower resting vmHRV (Grol and De Raedt, 2020). In a compassionate approach, this may facilitate emotional pain awareness, and subsequent decisions for helpful actions. Thereby, the individual is able to learn that negative information does not always translate into an aversive outcome (Borkovec et al., 2004), when the compassionate motivation is active (Gilbert, 2020).

In line with our hypothesis, the data evidenced significant greater HF-HRV increase after watching the video depicting the intentional actions of giving help, compared to video eliciting empathic sensitivity toward others’ suffering. Interestingly, most of the participants (88%) labeled the second video as more compassionate. This result replicates previous findings which identified increased vmHRV when individuals effectively engage in compassion interventions (Kim et al., 2020b), or improve their self-compassion competencies (Steffen et al., 2020). However, it also echoes recent evidence that both self-critical and self-compassionate writing were associated with a significant decrease in vmHRV during the task, but that only self-compassionate task produced a significant increase in vmHRV during recovery (Steffen et al., 2020).

Present data highlight that the engagement in compassion enables an appropriate autonomic response after seeing compassionate actions, indicative of how efficiently self-regulatory resources have been mobilized and used to overcome the emotional challenge and then return to resting level (Laborde et al., 2018). This quick return to parasympathetic response, called “vagal rebound” (Nederend et al., 2016), is crucial for therapy because, across time, it promotes an expansion of personal potential to self-regulate and react effectively, loosening resistances and blocks that in turn induce helplessness or shutdown states (Gilbert, 2020).

Our current findings suggest that compassion at first magnifies the saliency of emotional stimuli, consistent with the traditional function of this meditation (The Dalai Lama, 2001). Indeed, one aim of compassion focused training is to increase one’s sensitivity to the painful emotional experience of oneself and others, along with the courage and commitment to try to alleviate it (Gilbert, 2020). In fact, personal distress is supposed to surface during compassion focused training; that is why a key part of the training is to provide individuals with a grounding and soothing body routine (breathing and posture) and a psychoeducation that help them develop a de-shaming, non-judgmental, and self-reassuring stance toward suffering and one’s habitual patterns of emotional reactivity.

As to moderation analyses, a lower sensitivity to and motivation to engage with other’s suffering moderated the association between compassion for others and vmHRV recovery from video 1 (empathic stress condition), whereas this moderating role did not emerge for recovery from video 2 (compassionate action condition). Specifically, in the first condition compassion toward others was positively associated with HF-HRV recovery via lower self-compassionate engagement and action.

We explored changes in different affective states distinguishing between positive affect, low arousal negative affect, and high arousal negative affect. Results revealed a significant increase in low arousal negative affect and a parallel decrease in positive affect in response to video 1, whereas an increase in high arousal negative affect and parallel decrease in low arousal negative affect emerged in response to video 2. Recently, Gilbert and collaborators highlighted that kindness and compassion are associated with different emotions. Whereas kindness is generally associated with positive feelings, engaging in compassionate actions can give rise to a different emotional experience and affective states, mostly associated with anxiety, sadness, disgust, and anger (Gilbert et al., 2019). Consistently, the automatic analysis of spontaneous facial expressions in response to a short video eliciting compassion, showed that anger, disgust, sadness, and surprise occurred more often than fear, happiness, and contempt. In line with our results, anger occurred more often during compassion compared to baseline (Kanovský et al., 2020).

Several limitations must be considered when interpreting the current results. First, being the first to explicitly investigate vmHRV in association with the two subcomponents of compassion, the present study was intended to be preliminary and therefore conducted on a relatively small sample. Replication with a larger and more diverse sample is warranted before we can draw any conclusion on this issue. Moreover, there was an unequal sex distribution in our sample comprising of mostly females, and this may have biased the results. Indeed, differences in resting vmHRV have been well-documented (Koenig and Thayer, 2016), with females showing greater vagal activity, despite lower RR intervals. Likewise, sex differences emerge in the stronger negative relationship between resting vmHRV and empathic concern toward another in pain in women than in men (Tracy and Giummarra, 2017). Indeed, this disparity may be the consequence of different evolutionary selective pressures on females, fostering the evolution of mutual physiological connection between oxytocin and vagal functioning consistently with models of parental investment (Carter, 2014). However, recent meta-analytic results argue against the role of sex as a moderator of the association between (self- and other-oriented) compassion and vmHRV (Di Bello et al., 2020).

Lastly, the two videos were not randomly presented. In order to exclude the role of carry-over effects, we statistically compared resting 1 with resting 2 and we found that full recovery occurred before the beginning of video 2. However, we are aware that this is a serious methodological limitation that future studies should avoid.

Limitations notwithstanding, current results have potential clinical importance, contributing to inform our comprehension of the processes that are active when one engages in compassion, and although preliminary, highlight the importance of adopting a nuanced perspective on the complex physiological regulation that underlies compassionate responding to suffering. Encouraging finding suggests that this issue could be fruitfully explored by a time series analysis of the vmHRV signal, to disentangle how it fluctuates over the course of the empathic sensitivity condition and across the course of recovery (Kim et al., 2020a).

To conclude, compassion should not be seen as an antidote for negative affect, as it requires a dosage of personal suffering and pain before reaching its emotional and health benefits.

## Data Availability Statement

The raw data supporting the conclusions of this article will be made available by the authors, without undue reservation.

## Ethics Statement

The studies involving human participants were reviewed and approved by the IRB of Sapienza University of Rome. The patients/participants provided their written informed consent to participate in this study.

## Author Contributions

NP conceptualized and conducted the study. MD analyzed the data and wrote the initial draft of the manuscript. All authors contributed to the interpretation of the results, provided critical feedback, helped shape the analysis and manuscript, and approved the submitted manuscript.

## Conflict of Interest

The authors declare that the research was conducted in the absence of any commercial or financial relationships that could be construed as a potential conflict of interest.
